# Allele-specific copy number analysis of tumor samples with aneuploidy and tumor heterogeneity

**DOI:** 10.1186/gb-2011-12-10-r108

**Published:** 2011-10-24

**Authors:** Markus Rasmussen, Magnus Sundström, Hanna Göransson Kultima, Johan Botling, Patrick Micke, Helgi Birgisson, Bengt Glimelius, Anders Isaksson

**Affiliations:** 1 Science for Life Laboratory, Department of Medical Sciences, Uppsala University, Akademiska sjukhuset, SE-751 85 Uppsala, Sweden; 2 Department of Immunology, Genetics and Pathology, Uppsala University, Rudbeck Laboratory, SE-751 85 Uppsala, Sweden; 3 Department of Surgical Sciences, Uppsala University, Akademiska sjukhuset, SE-751 85 Uppsala, Sweden; 4 Department of Radiology, Oncology and Radiation Sciences, Uppsala University Akademiska sjukhuset, SE-751 85 Uppsala Sweden; 5 Department of Oncology and Pathology, Karolinska Institutet, SE-17177 Stockholm, Sweden

## Abstract

We describe a bioinformatic tool, Tumor Aberration Prediction Suite (TAPS), for the identification of allele-specific copy numbers in tumor samples using data from Affymetrix SNP arrays. It includes detailed visualization of genomic segment characteristics and iterative pattern recognition for copy number identification, and does not require patient-matched normal samples. TAPS can be used to identify chromosomal aberrations with high sensitivity even when the proportion of tumor cells is as low as 30%. Analysis of cancer samples indicates that TAPS is well suited to investigate samples with aneuploidy and tumor heterogeneity, which is commonly found in many types of solid tumors.

## Background

A characteristic feature of cancer cells is that their genomic DNA is altered [1]. Tumor cells frequently contain a wide range of aberrations, with gains, losses and translocations of genetic material often affecting a majority of the genome. In cancer, genomic aberrations are acquired during a process of tumor evolution, selecting for a tumor genome that provides growth and survival advantages over other cells.

The SNP array is currently one of the most efficient technologies for detecting copy number aberrations in tumor cells. SNP arrays measure allele-specific signals from SNP probes (A and B), allowing detection of both copy number alterations and allelic imbalances. High-density SNP arrays are available primarily on Affymetrix and Illumina platforms. Both platforms were originally developed for genotyping of diploid genomes. In order to use them for copy number analysis of tumor genomes, specialized bioinformatic tools are required. The main aim of such tools is the identification of boundaries and copy number for every aberration.

A commonly used strategy for identification of regions affected by genomic aberrations is segmentation of the total probe signals into genomic regions with similar average signal [2]. Conventional tools for copy number analysis only consider the total probe intensities relative to the average intensity of a set of (diploid) reference samples, usually called the Log-ratio [3]. Segments with an average Log-ratio near zero are often assumed to be copy number two, and any deviation beyond certain thresholds is called loss or gain accordingly.

The allele-specific copy number - that is, the total copy number and the specific number of copies of each original sister chromosome - can be determined from the allele-specific signals of the SNP markers. Several methods detect relative differences in total signals and allele-specific signal without determining absolute copy number [4,5]. One tool provides a manual interpretation of Affymetrix 500 K SNP array data, illustrated on glioblastomas [6]. An automated method for data from the Illumina platform has also been proposed [7]. PICNIC (Predicting Integral Copy Number in Cancer) can identify allele-specific copy numbers in cell lines and very pure tumor samples and is designed for Affymetrix SNP 6.0 arrays [8]. However, tumor samples frequently suffer from an admixture of genetically normal cells. The effects of normal cell content on probe and SNP intensities is significant and must be taken into account when analyzing most tumor samples. Göransson *et al. *[5] proposed the CNNLOH Quantifier to estimate the proportion of normal cells and copy-number-neutral loss of heterozygosity (LOH) from allele frequency. GAP (Genome Alteration Print), OncoSNP and ASCAT (Allele-Specific Copy number Analysis of Tumors) have been developed for allele-specific copy number analysis of Illumina SNP array data [9-11]. TumorBoost allele-specific signal normalization is suitable for Affymetrix SNP arrays with paired normal samples [12]. Methods developed for Illumina SNP arrays are not directly applicable to data from Affymetrix arrays due to different signal and noise characteristics. PSCN (Parent-Specific Copy Number) has recently been published as a platform-independent solution and was shown to outperform several frequently used copy number analysis tools on a dilution data set based on a diploid tumor sample [13].

### Tumor ploidy

It is well known that many types of tumors frequently have genomic aberrations involving gain or loss of whole or large parts of chromosomes. Thus, the average ploidy or total genomic content of tumor cells cannot be assumed to be 2N. A fixed amount of genomic DNA is hybridized to the array (rather than a fixed number of cells), and the basic normalization procedure includes median-centering of the total probe intensities. Conventional microarray copy number analysis is based on comparing the probe intensities to those of a set of diploid reference samples. This works well for detecting aberrations in diploid non-cancer samples as the normalized intensity of copy number two should coincide for query and reference data. It may also work reasonably well on tumors that have few and relatively small genomic aberrations. However, many individual tumors have such extensive genomic aberrations that the assumption that the query cells have a genomic content of 2N on average is severely violated. This can lead to systematic misidentification of copy numbers throughout the entire sample, by one or more copies [8].

### Tumor heterogeneity

Tumor samples are a mix of cancer cells and genetically normal cells. The proportion of tumor cells can vary considerably, complicating the analysis since the measured signal from any locus will be a combined signal from both tumor and non-tumor cells. If the proportion of tumor cells is too low, aberrations will remain undetected. Comparisons of detected aberrations in crude tumor samples with those detected in microdissected tumor cells from the same original samples have shown that many copy number aberrations are overlooked even in relatively pure tumor samples [5].

### Tumor cell heterogeneity

Some copy number alterations are variable in nature, their genomic content prone to repeated duplication or deletion in future cell generations [14]. In addition, copy number aberrations in tumor cells may arise several times throughout tumor development, and may give rise to different subclones. New aberrations may change the proliferative activity of that cell and its progeny, possibly making them constitute a growing proportion and eventually a majority of the tumor cells. The extent of the proliferative advantage and the time between occurrence of the aberration and tumor collection influence the proportion of tumor cells with each aberration [15]. As a consequence of the tumor cell heterogeneity, the average copy numbers of heterogenic genomic regions may be non-integer. Long non-integer regions may severely disturb a model-based copy number analysis as they do not fit the pre-determined relationship between signal and copy number.

### Tumor Aberration Prediction Suite

We present a tool and algorithm for allele-specific copy number analysis of tumor samples on Affymetrix 500 K and SNP 6.0 arrays called Tumor Aberration Prediction Suite (TAPS). It handles samples with aneuploidy and the presence of normal cells and facilitates detection of tumor cell heterogeneity. We describe allele-specific copy number profiling of 7 lung cancer cell lines and 12 colon tumor samples, partially validated through spectral karyotyping (SKY) and DNA ploidy analysis.

## Results and discussion

**Figure 1:**
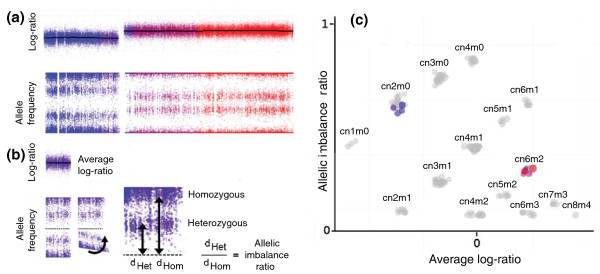
**Schematic illustration of the Tumor Aberration Prediction Suite **(**TAPS) method for visualization of chromosomal aberrations in tumor samples**. A scatter plot of the average Log-ratio and the allelic imbalance ratio of all segments in a sample reveals the aberrations present. **(a) **The example chromosome is segmented with respect to Log-ratio and allele frequency. **(b) **In the allele frequency pattern, the probes' distance from heterozygosity (equal signal from alleles A and B) is clustered on two means, representing heterozygous (d_het_) and homozygous (d_hom_) SNPs. The allelic imbalance ratio d_het_/d_hom _ranges from near zero with equal allelic copy numbers and low noise to near one with loss-of-heterozygosity (LOH), low normal cell contamination and low noise. **(c) **Schematic illustration of a Log-ratio/allelic imbalance ratio scatter plot. An example region in blue can be identified as cn2m0, that is, copy number 2 and minor copy number 0 (LOH). Each object in the scatter plot corresponds to a chromosomal segment. Segments are colored according to their chromosomal position (a) and segments on other chromosomes of the same sample are plotted in grey. The number of possible variants of allele-specific copy number increases with the total copy number. Lower copy numbers are more affected by noise and normal cell contamination, which reduce any allelic imbalance. Deletions and copy number neutral LOH may therefore show less allelic imbalance than high copy numbers that retain one or more copies of its minor allele. Note how variants with the same minor copy number tend to line up diagonally, facilitating the interpretation.

We developed TAPS as a tool for investigation and identification of allele-specific copy numbers from Affymetrix SNP array data. TAPS handles samples with aneuploidy and significant normal cell content, and facilitates detection of several kinds of tumor cell heterogeneity. Raw array data are first normalized and segmented by conventional means (see Materials and methods). TAPS visualizes and estimates allele-specific copy number of the genomic segments based on their average probe intensity (Log-ratio) and their allelic imbalance ratio (see Materials and methods). The allelic imbalance ratio is sensitive to signal differences in heterozygous SNPs in a genomic region, and is robust enough to distinguish different allele-specific copy number variants based on small changes in allele-specific signals (Figure 1b). It is particularly useful as it does not require heterozygous (informative) SNPs to be known in advance or to be estimated through an allele frequency cutoff. TAPS is available from the authors [16].

All aberrations in a single sample are visualized by plotting the allelic imbalance ratio against the average Log-ratio for the genomic segments. Segments group into clusters where the relative positions of the clusters provide the basis for manual or automatic allele-specific copy number calls (Figure 1c). Highlighting separate chromosomes or arbitrary segments in this environment shows their relevant characteristics relative to the whole sample, creating the basis for correct total and minor allele copy number calls. This is especially useful when tumor cell heterogeneity or normal cell content would complicate the analysis.

### The current state of tumor copy number analysis

A number of recent bioinformatic methods use allele-specific information to take aneuploidy or non-tumor cells into account in detecting genomic aberrations. Due to the different signal and noise characteristics of microarray platforms, most methods are designed to work on array data from one platform only. Some specialize on cell lines, handling aneuploidy well on very pure cancer samples but without taking normal cell content into account [8]. Recent methods that model the contributions from aneuploidy and non-tumor cells at the same time are GAP, ASCAT and PSCN [9,11,13]. These methods require identification of individual informative SNP markers that are heterozygous in non-tumor cells, either from the tumor sample data or from genotyping matched non-tumor tissue samples on a separate array. TAPS uses a clustering solution to estimate the relationship between heterozygous and homozygous SNPs, and requires no clear separation between them. GAP and ASCAT perform better than the previous generation of methods. However, at least for some notoriously difficult types of tumors such as breast cancer, a considerable fraction (19%) of tumors does not fit the ASCAT models well enough to be analyzed [11]. In addition, samples tend not to fit these models beyond copy number 4 to 6 [9,11]. Reasons for limited performance may be that some factors affecting the array intensities are unknown, that certain copy number variants required by the model are missing, and that tumor cell heterogeneity prevents the data from fitting the models. The result is that many samples can either not be analyzed or the prediction of allele-specific copy numbers will be incorrect.

### Performance on low tumor cell content

**Figure 2:**
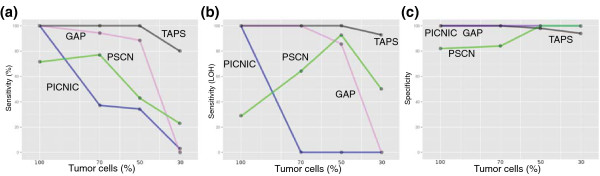
**Performance of TAPS and three other methods in identifying aberrations influenced by normal cell content**. The performace of TAPS is compared to three other methods, PICNIC, PSCN and GAP. **(a) **Sensitivity as the percentage of correctly identified out of 35 large aberrations. **(b) **Sensitivity in the subset of (a) with loss of heterozygosity (*n *= 14). **(c) **Specificity as the percentage of unaltered genome that was correctly reported as unaltered. All methods showed high specificity, partially due to some of them reporting everything as unchanged at low tumor cell content.

TAPS was compared to three other recently developed tools for allele-specific copy number analysis of SNP6 arrays: PICNIC, GAP and PSCN [8,9,13]. To evaluate performance on samples with normal cell contamination, we prepared a dilution series by mixing DNA from lung cancer cell line H1395 and its patient-matched blood cell line BL1395 (with normal karyotype). Four samples with normal cell proportions ranging from 30% to 100% were analyzed on SNP6 arrays and the resulting data subjected to copy number analysis with the four methods. Overall sensitivity is shown in Figure 2a and performance on regions with LOH is shown in Figure 2b. With high tumor content, aberrations with and without LOH were well detected by TAPS and GAP. PICNIC, which is designed for cell lines, proved excellent at 100% tumor cells but vulnerable to normal cell content. TAPS performed particularly well with a low proportion of tumor cells, demonstrating high sensitivity at 30% tumor cells. The performance of PSCN was similar except that it reported incorrect total copy number (though finding LOH) of some aberrations with LOH at high tumor cell content. All four methods showed impressive specificity (Figure 2c). Note, however, that PICNIC and GAP reported most or all of the genome as unaltered at 30% tumor cells. Raw data and copy number output are available at the Gene Expression Omnibus (GEO) with accession number [GEO:GSE29172]. TAPS scatter plots visualizing copy numbers at different tumor cell proportions are available in Additional file 1.

### Chromosomal aberrations in lung cancer cell lines

**Figure 3:**
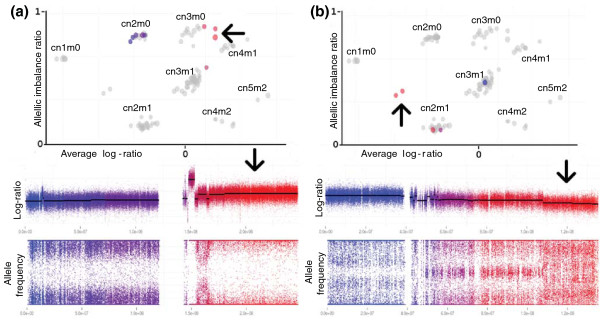
**Identification of heterogeneity within the tumor cell population**. Plotting a chromosome highlighted against the whole sample allows discovery of genomic regions with heterogeneity within the tumor cell population, shown here on two regions in lung cancer cell line H1395. The arrows mark the chromosomal segments and their respective positions in the scatter plots. **(a) **On chromosome 1, most of the q arm has loss of heterozygosity and an average of about 3.5 copies. The high-copy segment near the centromere is not visible in this scatter plot. **(b) **The end of 10q is seen between the clusters for normal ploidy and deletion, indicating deletion only in part of the cell population.

To evaluate the performance of TAPS on samples with varying ploidy, we retrieved published SKY karyotypes and Affymetrix 250 K array data [GEO:GSE17247] for seven lung cancer cell lines [17,18]. For each sample, we performed segmentation of the genome with respect to allele-specific intensities. We then visualized each segment relative to the full-sample background using TAPS. Five samples were found to have an average copy number above 2.5. In all seven we were able to validate the average ploidy with the SKY karyotypes. The SKY karyotype of cell line H1395 reported two heterogeneous aberrations where about 50% of the cells would carry each variant. We cultured H1395 cells and analyzed them on a SNP6 array to improve data quality and resolution. We could clearly observe the expected tumor cell heterogeneity for both loci, one with a mix of normal ploidy and a specific aberration, and one with a mix of two different aberrations (Figure 3a, b).

#### Variable copy numbers and double minutes

**Figure 4:**
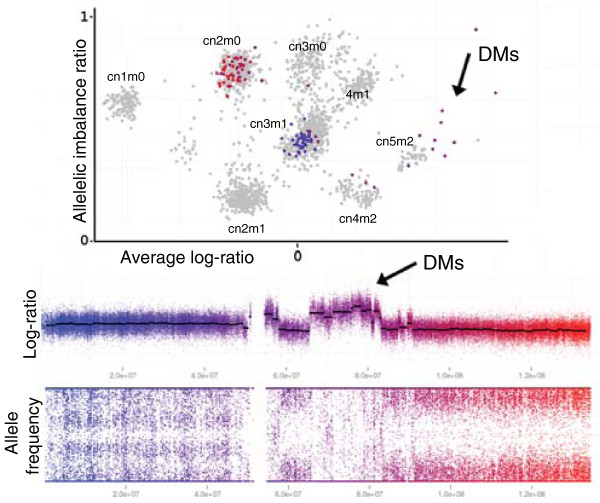
**Double minute chromosomes in a lung cancer cell line**. A short-segment TAPS scatter plot illustrates the origin of double minute chromosomes (DMs). Lung cancer cell line H1395 has three copies of chromosome arm 11p (blue) and no loss of heterozygosity (LOH). Most of 11q is copy number 2 with LOH (red). The DMs appear as several successive short segments with varying total copy numbers (violet). Their minor allele varies between zero and two copies when the major allele has two copies, and remains at two when the major allele is above two. This suggests that circular DMs originate from the sister chromosome that in most of 11q has been lost. The remaining sister chromosome is duplicated but otherwise unchanged.

The automatic copy number analysis of TAPS does not predict all copy number intensities from a single mathematical model. Instead the intensities observed for lower copy numbers are used iteratively to predict those of higher copy numbers. On short aberrations, noise may cause segmentation errors, making the exact copy number hard to determine. This is of particular importance for genomic aberrations such as double minutes (DMs), as their numbers vary greatly between cells and the observed copy number reflects an average [14]. To this end, TAPS copy number analysis outputs an additional set of short-segment scatter plots, where the segments produced by circular binary segmentation (CBS) have been further segmented into regions of 200 to 400 markers. On such a plot of chromosome 11 from H1395, a region on the q arm displays several short copy number aberrations afflicting only one of the original sister chromosomes (Figure 4). SKY karyotyping of the cell line has previously identified DMs from chromosome 11 [18]. This region is further illustrated at different tumor cell concentrations in Additional file 1. The allelic imbalance ratio remains sensitive to small differences in allele frequency for very short segments, and TAPS is therefore suitable for investigating this particular form of tumor heterogeneity.

A key to a thorough analysis with TAPS is the visualization of samples, which allows the researcher to assess widespread tumor heterogeneity, average ploidy and normal cell content. This visual inspection allows the researcher to identify samples that may be problematic due to frequent tumor cell heterogeneity, very low tumor cell content or poor quality. Such samples can usually be handled by the automatic copy number analysis, but some manual input may be required.

### Chromosomal aberrations in colorectal cancer tumor samples

**Figure 5:**
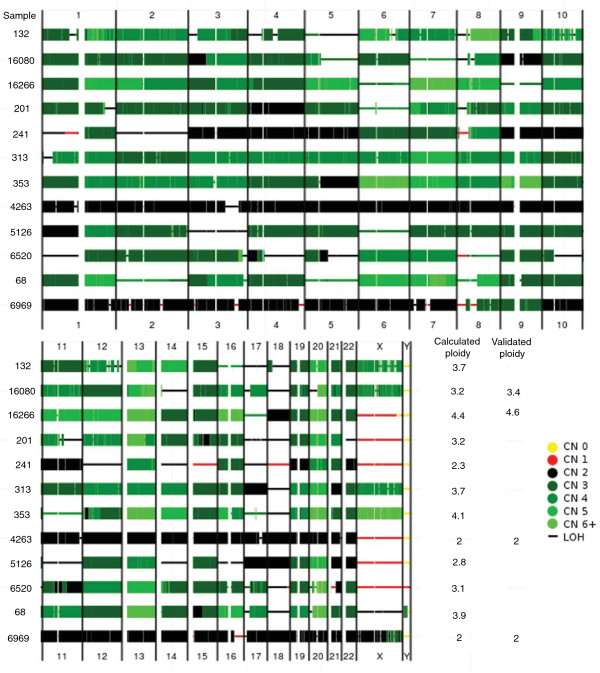
**Allele-specific copy number aberrations and ploidy in 12 colon cancer samples**. Genome-wide overview of copy numbers (CN) determined by TAPS are indicated using color (see key in figure). In addition, loss of heterozygosity (LOH) is indicated using thin lines. Note the extensive presence of LOH in many samples, often coinciding with high copy numbers. Average ploidy of tumor cells calculated using TAPS and through DNA ploidy analysis.

Twelve colorectal cancer tissue samples analyzed on Affymetrix SNP 6.0 arrays were used to evaluate the performance of TAPS on tumor samples. Allele-specific copy numbers were produced using TAPS automated calling. Four samples, two with predicted aneuploidy, were selected for DNA ploidy analysis (see Materials and methods) as an independent measure of the average ploidy of the tumor cells. Total copy numbers and LOH, and computed and independently measured average ploidy for all 12 samples are shown in Figure 5. The high correspondence between the average copy number in the tumor cells obtained by TAPS and DNA ploidy analysis indicates that TAPS is highly suitable for analyzing tumor samples.

### Limitations

TAPS has been developed and validated for SNP array data from Affymetrix 500 K and SNP 6.0 arrays. As long as allele-specific signals are not subjected to processing meant only for genotyping of diploid samples, we expect the TAPS approach to work well on data from other platforms. With the different noise characteristics and data processing of non-Affymetrix microarrays, automatic copy number calling with TAPS should not be expected to work without modification.

Before copy number analysis in TAPS, raw intensity data should be normalized and segmented. We found a wide selection of tools (including Affymetrix Power Tools, AROMA and Biodiscovery/Nexus Copy Number) to perform satisfactory normalization and can thus be used with TAPS. For direct use of .CEL files a wrapper that uses AROMA CRMAv2 [19] to process raw intensities is available with TAPS. Segmentation is normally performed either with a hidden Markov model (HMM) or with CBS, the latter of which is available in R through the DNACopy package as well as in Nexus and other software. While a HMM is generally capable of producing fewer incorrect segment breaks than CBS, it requires a very good prior estimate of the signal-copy number relationship to do so. Moreover, tumor cell heterogeneities frequently manifest as non-integer copy numbers, causing a HMM segmentation to oscillate almost randomly between the nearest integer copy numbers. We found the CBS segmentation approach to be much more suitable for tumor data, and recommend it for use with TAPS.

Molecular methods that analyze extracts from tumor tissue have general limitations. One problem is that the heterogeneity of tumor cells has the consequence that unless the entire tumor is used for the molecular analysis, there will be a sampling of the tumor cells. The extracted DNA will thus be more or less representative of the tumor cells of the tumor.

In the colon cancer material, DNA was extracted from sections of the tumor tissue, achieving a reasonable, albeit not perfect, representation of the tumor. Thus, additional genomic aberrations may therefore be present in the tumor cells that may be missed due to insufficient sampling. However, since relatively little tumor cell heterogeneity is detected in the material that was sampled, we believe that genomic aberrations present in a major proportion of the tumor cells rarely remain undetected.

Improved bioinformatic tools are important for analysis of tumor samples on SNP microarrays. Consequences of poor performance of such tools include systematically underestimating copy numbers in hyperploid tumors, false detection of LOH where the minor copy number is relatively low, and failure to detect aberrations at all due to the presence of normal cells that weaken the signal from tumor cells. Improved data analysis may become a decisive factor that improves the overall analysis to allow the discovery of significantly new genomic factors with important roles in tumor development.

A new generation of large-scale DNA sequencing technologies is starting to be applied to tumor samples [15,20,21]. It should be pointed out that the issues relevant for analyzing tumors using SNP microarrays are equally relevant for sequencing technologies. Methods such as TAPS need to be developed for sequencing data and will allow correct identification of genomic aberrations in tumor samples analyzed by sequencing.

## Conclusions

TAPS provides reliable information on allele-specific copy number in tumor samples analyzed on Affymetrix arrays, without the need for matched normal samples. The use of TAPS may help in elucidating critical events in tumor development that could affect care and management of cancer patients.

## Materials and methods

### Allelic imbalance ratio

TAPS uses the B-allele frequencies (BAF), defined as (B/(A + B)), where A and B are the normalized intensities of the A and B probes, to calculate the allelic imbalance ratio of genomic segments. TAPS takes the absolute values of BAF - 0.5 (the distance to equal A and B signal, for each SNP) and clusters on two means, representing heterozygous and homozygous SNPs. The allelic imbalance ratio is produced by dividing the inner cluster center by the outer. The resulting value will be close to zero in cases of a balanced copy number variant (usually about 0.1 due to forcing two means, and the effects of noise), and similarly close to one in cases of a very unbalanced copy number variant (such as a high copy number with LOH) and very low normal cell content.

### Copy number visualization

For each segment, TAPS considers the mean Log-ratio of all probes and the allelic imbalance ratio of the SNPs. The mean Log-ratio reflects the total copy number of the segment. The allelic imbalance ratio reflects the relationship between the alleles. However, with an unknown average ploidy of tumor cells and an unknown proportion of normal cells, the exact relationship between Log-ratio, allelic imbalance ratio and the allele-specific copy numbers of the tumor cells will vary between samples.

To visualize the tumor aberrations in a sample, Log-ratio is plotted against allelic imbalance ratio for all segments. A high proportion of normal cells reduces the allelic imbalance caused by imbalanced tumor aberrations, and the effect on Log-ratio of total copy number changes. However, segments will still appear in a predictable fashion with respect to one another, and a good assessment can be made with as little as 30% tumor cells (Figure 3).

### Copy number calling

TAPS includes an algorithm for automatic estimation of total and minor copy number. It first estimates the (sample-specific) relationship between Log-ratio, allelic imbalance and copy numbers. This crucial step can be assisted by a visual interpretation of the TAPS scatter plots. The calling algorithm implemented in TAPS then uses the Log-ratio and allelic imbalance ratio of lower copy numbers to estimate the characteristics of higher copy numbers. By iteratively working from lower to higher copy numbers, TAPS continuously adjusts expectations according to observations. TAPS is available from the authors as extensively commented R code. A simplified overview is presented here.

Step 1: estimate the Log-ratio of copy number two, using the Log-ratio and allelic imbalance ratio of the lowest-intensity long autosomal segments. The relatively low allelic imbalance of unaltered regions compared to LOH and single-copy gains and losses is the best indicator of copy number 2.

Step 2: find the allelic imbalance ratio of cn1, cn2m1 (2 with minor copy number 1) and cn2m0 (2 with minor copy number 0, that is, LOH) from all segments belonging to copy numbers 1 and 2.

Step 3: if step 1 or 2 fails, the analyst may supply an initial interpretation from a TAPS scatter plot.

Step 4: for each successive higher copy number, use the difference in Log-ratio between lower copy numbers to estimate its Log-ratio. Set it to the median of any segments that match the expectation well (note that segments are weighted on their length). If no such segments exist, set it to the expectation. The Log-ratio difference between successively higher copy numbers tends to drop slowly but steadily, and this way TAPS adjusts its expectations according to observations in the current sample.

Step 5: at copy number 3 and higher, use the differences in allelic imbalance ratio seen on lower copy number variants (such as cn1, cn2m1 and cn2m0 for copy number 3) to predict the allelic imbalance ratio of copy number variants (such as cn3m1 and cn3m0). Set them to the median of any segments of the correct copy number that closely match the expectation (note that segments are weighted on their length). If no such segments exist, set it to the expectation. This step uses the tendency of copy number variants with the same minor copy number to line up diagonally (with a slowly decreasing slope), which can be seen in the TAPS scatter plots.

### Sample preparation and microarray experiments

Twelve colon cancer samples were selected from a set of immediately frozen tumor biopsies from patients operated upon for a colorectal cancer at the hospitals in Uppsala or Västerås, Sweden. Two of the 12 had appeared to fit a conventional copy number analysis well, while the remaining 10 had raised suspicions of hyperploidy. All patients gave informed consent according to the research ethical committee at Uppsala University for the storage, isolation of DNA and use of the material in research projects. The tumor cell content in each sample was at least 50% based upon an examination by a pathologist (JB or PM) of a hematoxylin-eosin stained section. All patients had stage II and III colon cancer. All samples were fully anonymized. DNA was extracted from two to ten frozen tissue sections (10 μm) using the QIAamp DNA Mini Kit (Qiagen, Hilden, Germany). DNA concentrations were measured with a ND-1000 spectrophotometer (NanoDrop Technologies, Wilmington, DE, USA).

Lung cancer cell line H1395 and patient-matched blood cell line BL1395 were obtained from the American Type Culture Collection (ATCC) and cultured according to their recommendations. DNA extraction was performed using the DNeasy Tissue Kit (Qiagen). Dilutions representing 30, 50 and 70% tumor cell content were prepared from the extracted DNA. The higher (near-triploid) DNA content of the tumor cells was compensated for by using 42, 65 and 80% tumor DNA.

Array experiments were performed according to the standard protocols for Affymetrix Genome-Wide Human SNP Array 6.0 arrays (Cytogenetics Copy Number Assay User Guide, P/N 702607 Rev2), Affymetrix Inc., Santa Clara, CA, USA). Quality control was performed in Affymetrix Genotyping Console version 3.0. Array data, including the cell line and colorectal tumor copy numbers, are available at the GEO [GEO:GSE26302].

### Data preparation and analysis

Raw data (.CEL files) from colorectal cancer samples were normalized, Log-ratio and allele frequency was extracted and segmentation was performed in BioDiscovery Nexus Copy Number 3.0 with European HapMap samples as a reference set and using the Rank Segmentation algorithm based on CBS. Downstream analysis was performed in R using the TAPS suite, including allelic imbalance ratio calculation, plotting and copy number calling.

Published lung cancer cell line raw data (Affymetrix GeneChip Human Mapping 250 K) were processed in BioDiscovery Nexus Copy Number 3.0 with European HapMap samples as a reference set and using the Rank Segmentation algorithm. Downstream analysis of Log-ratio, allele frequency and segments was performed in R using the TAPS suite. The average copy number of each sample was read from the TAPS scatter plots. SKY karyotypes from samples H2122, H2126, H1395, H1437, H1770, H2087 and H2009 were downloaded and used to verify the result of TAPS [18]. Summaries of the analysis are available in Additional file 2.

### Comparative analysis

Copy number analysis with PICNIC, GAP, PSCN and TAPS was performed on SNP6 raw data from the three diluted samples (30, 50 and 70% tumor cells) and the pure H1395 cell line. We selected all aberrations on which the allele-specific copy number calls of at least three of the four methods coincided for the all-tumor-cell sample. These were 35 large regions, representative of all types of copy number aberrations in the sample, and covered the majority of the genome. We then observed whether the four methods, for each region, gave matching copy number calls in the normal cell-diluted samples. Sensitivity was calculated as percentage of the 35 aberrations that were mostly correct (correct allele-specific copy number in more than half of that region). Since different segmentation strategies are used by the different methods, exact breakpoints were not considered important. Specificity was measured by first defining the truly unaltered genome using the pure tumor sample and concurring (heterozygous copy number 2) calls of at least three methods. We then summed up, for the four methods and the diluted samples, the percentage of the truly unaltered genome (True negatives + False positives) that were reported as such (True negatives), applying the general definition of specificity as True negatives/(True negatives + False positives). Automatic copy number analysis was used with all methods.

### DNA ploidy analysis

Formalin-fixated, paraffin-embedded tissues corresponding to four of the colorectal cancer tissue samples were deparaffinized and analyzed for DNA content as previously described [22].

## Abbreviations

ASCAT: Allele-Specific Copy Number Analysis of Tumors; BAF: B-allele frequency; CBS: circular binary segmentation; DM: double minute; GAP: Genome Alteration Print; GEO: Gene Expression Omnibus; HMM: hidden Markov model; LOH: loss of heterozygosity; PICNIC: Predicting Integral Copy Number in Cancer; PSCN: Parent-Specific Copy Number; SKY: spectral karyotyping; SNP: single nucleotide polymorphism; TAPS: Tumor Aberration Prediction Suite.

## Competing interests

The authors declare that they have no competing interests.

## Authors' contributions

HB and BG provided tumor samples for the study. PM and JB performed pathological examination of tumor samples. AI conceived the study and wrote the manuscript. HGK participated in its design and coordination. MR conceived and developed the TAPS method, conceived the study and wrote the manuscript. MS supervised DNA preparation and copy number validation. All authors read and approved the final manuscript for publication.

## Supplementary Material

Additional file 1 **Dilution series summary**. This file illustrates the sensitivity of TAPS using short-segment scatter plots of lung cancer cell line H1395, for several different tumor cell concentrations. Click here for file

Additional file 2 **Lung cancer cell line summaries**. This file contains TAPS scatter plots illustrating the copy number analysis result of the seven lung cancer cell lines, and matching SKY karyotypes for comparison. Click here for file
