# Supplementary material for: Allele-specific copy number analysis of tumor samples with aneuploidy and tumor heterogeneity
Source: Genome Biol. 2011 Oct 24;12(10):R108. doi: 10.1186/gb-2011-12-10-r108 (PMC3333778; doi:10.1186/gb-2011-12-10-r108)

UPPSALA
UNIVERSITET

Short-segment TAPS scatter plots of chromosome 11 of lung cancer cell line H1395 at different tumor cell concentration levels

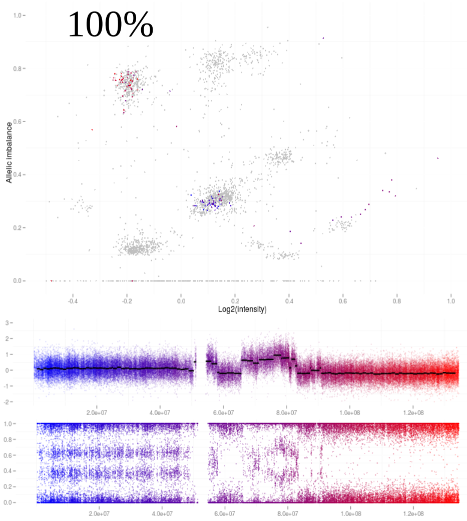

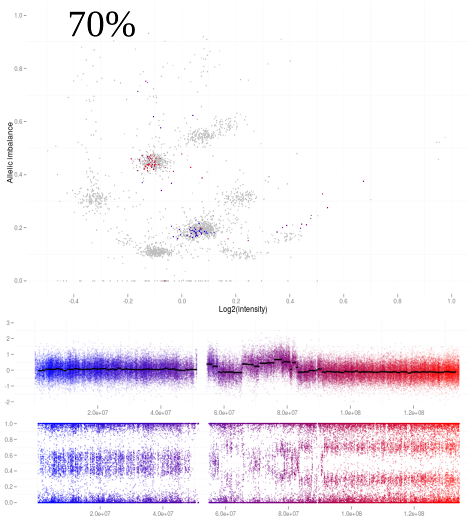

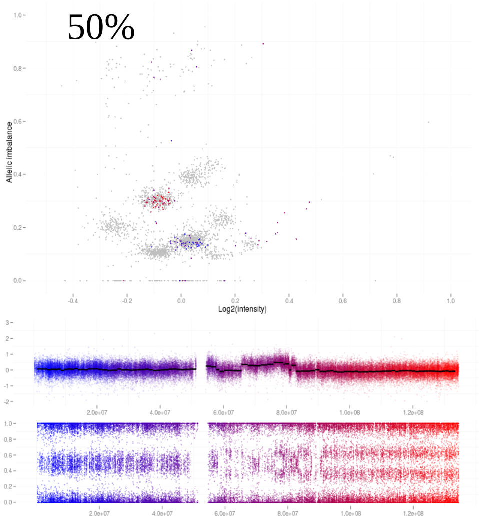

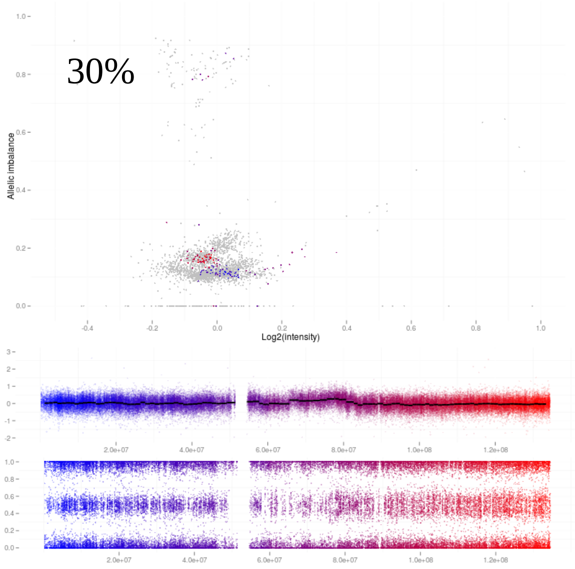

Scatter plots form the foundation of TAPS visual or automatic interpretation of copy numbers. The p arm (blue) has a total of 3 copies, and a minor allele copy number of one (two of one, and one of the other originial 11p chromosome arm). Most of the q arm (red) has lost one original sister chromosome and duplicated the other (LOH), but throughout its first half (violet), parts of the deleted chromosome arm remain at variable copy numbers. These characteristics atr typical for double minute chromosomes, and are discernable using TAPS with as little as 30% tumor cells.

Short-segment TAPS scatter plots of chromosome 2 of lung cancer cell line H1395 at different tumor cell concentration levels

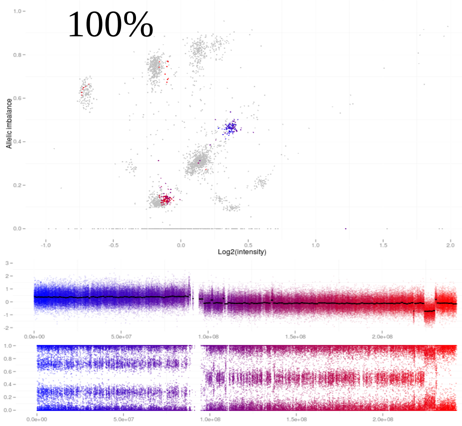

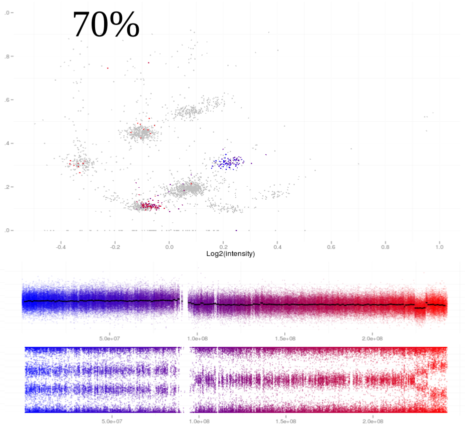

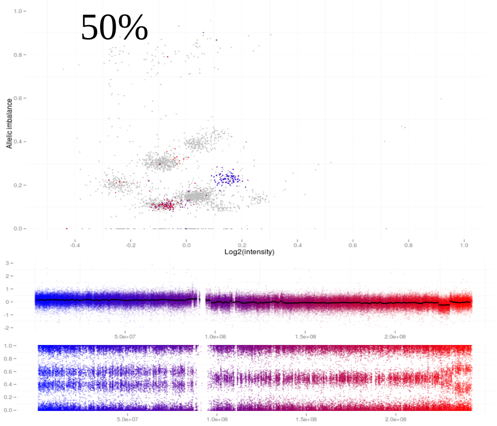

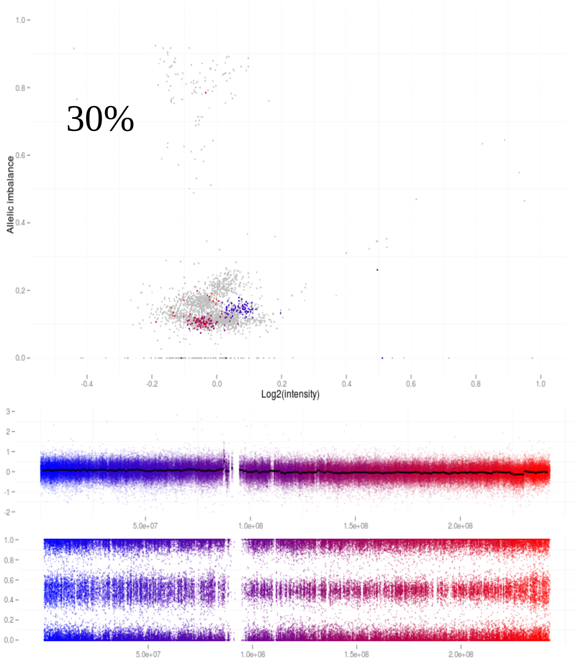

The p arm has a total of 4 copies, and a minor allele copy number of one (three of one plus one of the other originial 2p chromosome arm). Most of the q arm is unaltered, but a deleted segment and a segment with LOH can be seen at the end. These copy numbers are discernable at as little as 30% tumor cells.

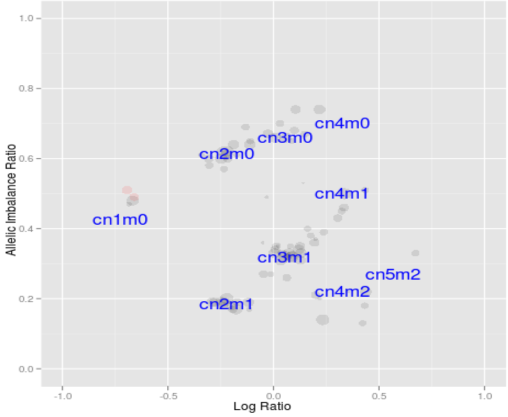

Supplement: Additional file 1 — Dilution series summary. This file illustrates the sensitivity of TAPS using short-segment scatter plots of lung cancer cell line H1395, for several different tumor cell concentrations. [file gb-2011-12-10-r108-S1.PDF]
