# Supplementary material for: Allele-specific copy number analysis of tumor samples with aneuploidy and tumor heterogeneity
Source: Genome Biol. 2011 Oct 24;12(10):R108. doi: 10.1186/gb-2011-12-10-r108 (PMC3333778; doi:10.1186/gb-2011-12-10-r108)

UPPSALA UNIVERSITET

H2122

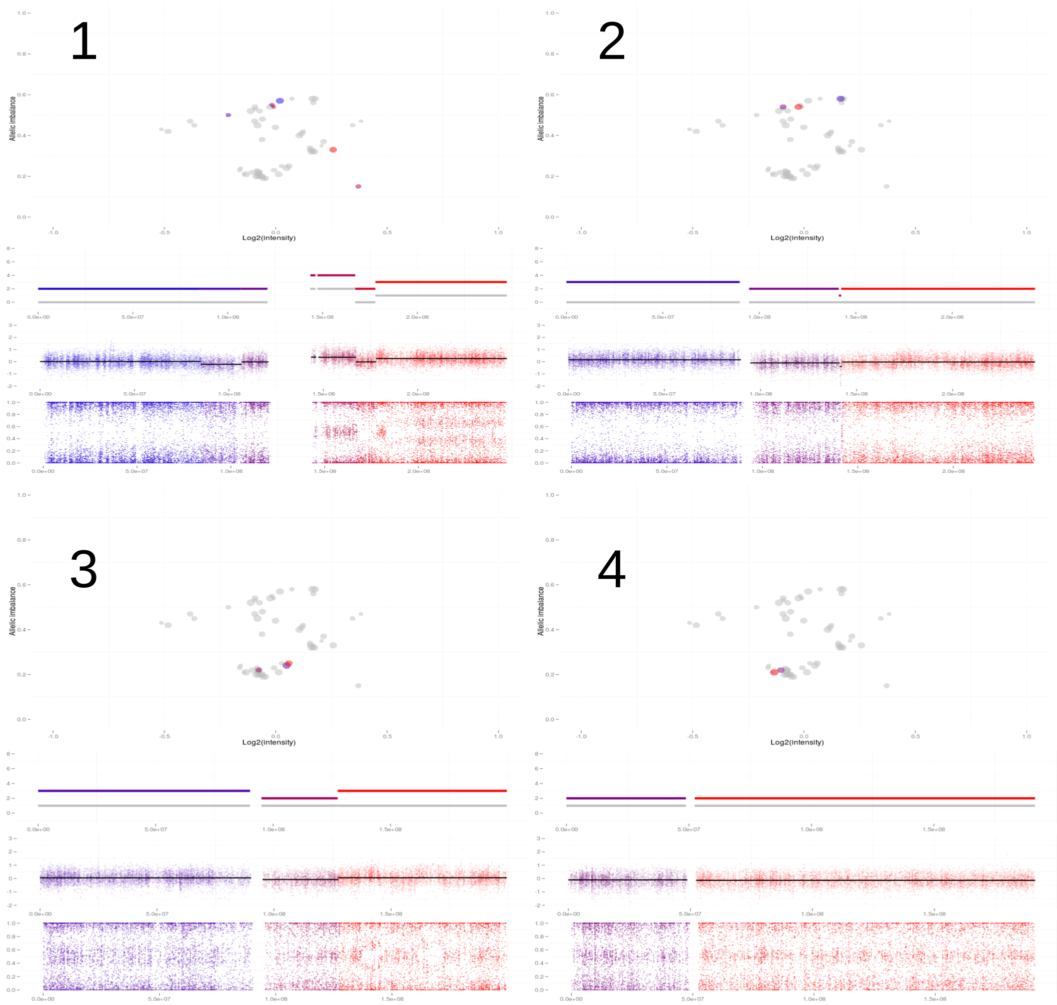

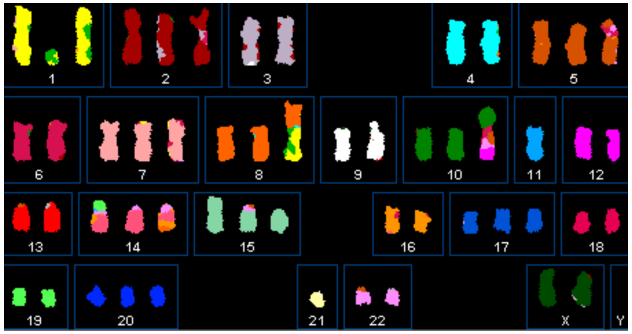

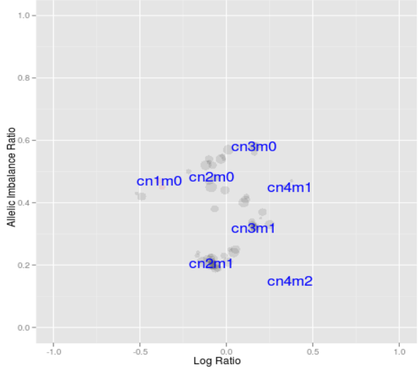

Lung cancer cell line H2122. TAPS scatter plots of chromosomes 1-4 display the segments of the current chromosome (color-coded blue to red) in relation to all other segments thoughout the sample (grey dots). The Log-ratio and Allelic Imbalance Ratio corresponding to allele-specific copy numbers estimated by TAPS is displayed in the summary plot below. Copy numbers correspond well to the SKY karyotype. Note however that most of chromosome 3 was assigned three copies TAPS, and two copies according to SKY. A closer look at the scatter plot of chromosome 3 puts these segments between copy numbers 2 and 3, indicating some heterogeneity.

UPPSALA UNIVERSITET

H2126

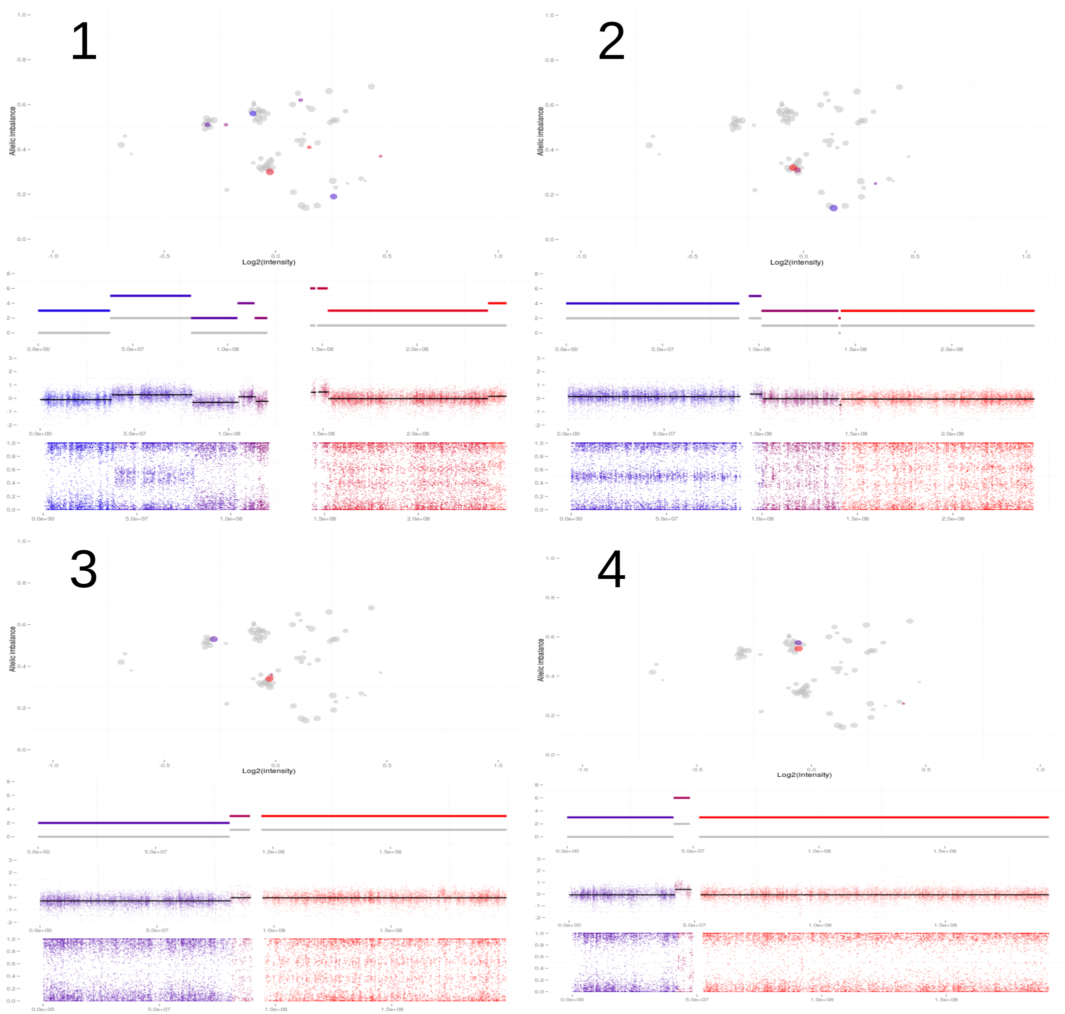

Lung cancer cell line H2126. TAPS scatter plots of chromosomes 1-4 display the segments of the current chromosome (color-coded blue to red) in relation to all other segments thoughout the sample (grey dots). The Log-ratio and Allelic Imbalance Ratio corresponding to allele-specific copy numbers estimated by TAPS is displayed in the summary plot below. Copy numbers correspond well to the SKY karyotype. Note that the SKY karyotype does not tell which region of a chromosome that has been altered.

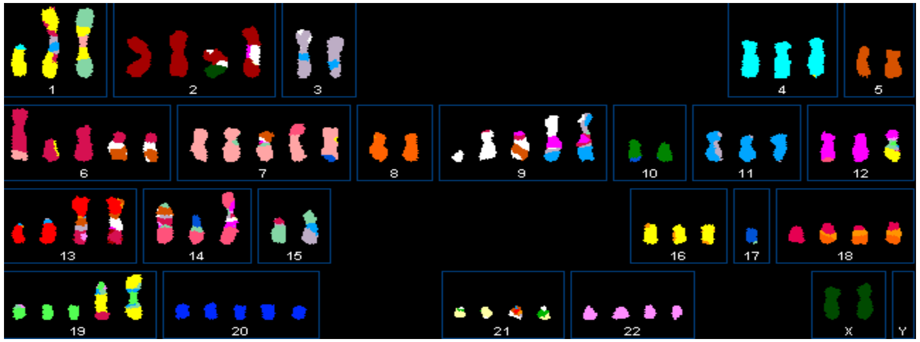

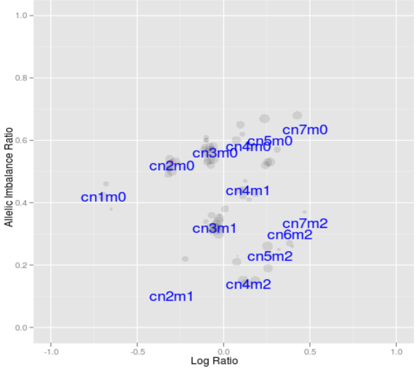

UPPSALA
UNIVERSITET

H1395

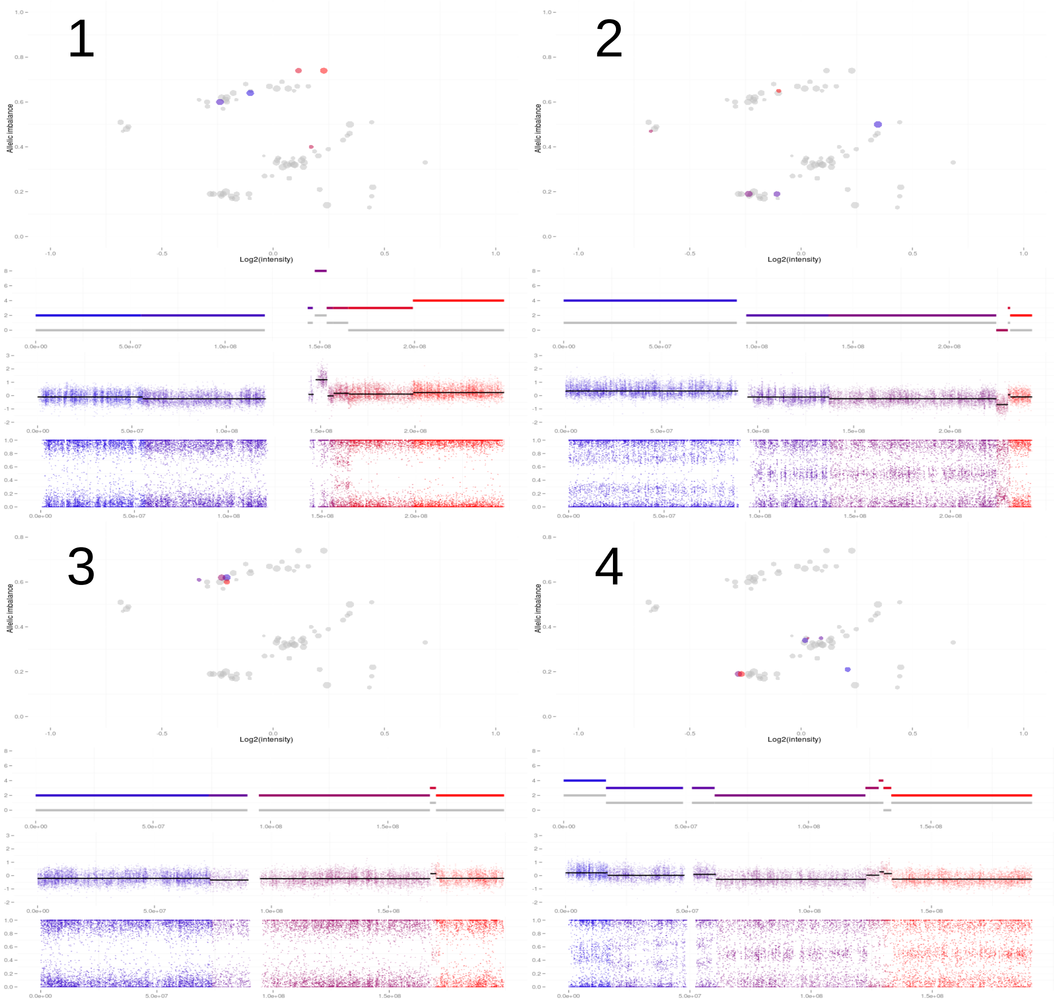

Lung cancer cell line H1395. TAPS scatter plots of chromosomes 1-4 display the segments of the current chromosome (color-coded blue to red) in relation to all other segments thoughout the sample (grey dots). The Log-ratio and Allelic Imbalance Ratio corresponding to allele-specific copy numbers estimated by TAPS is displayed in the summary plot below. Copy numbers correspond well to the SKY karyotype. Note that a part of chromosome 2 has fused with chromosome 20.

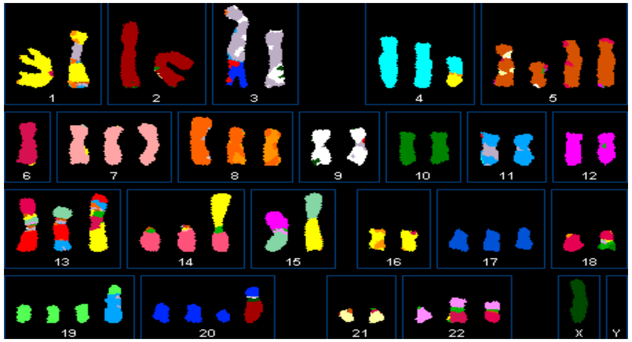

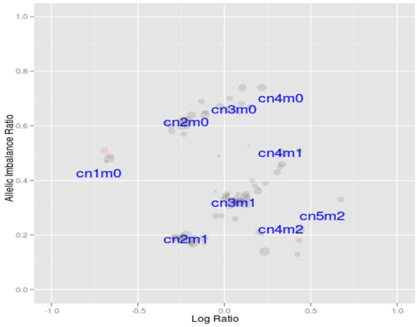

UPPSALA
UNIVERSITET

H1437

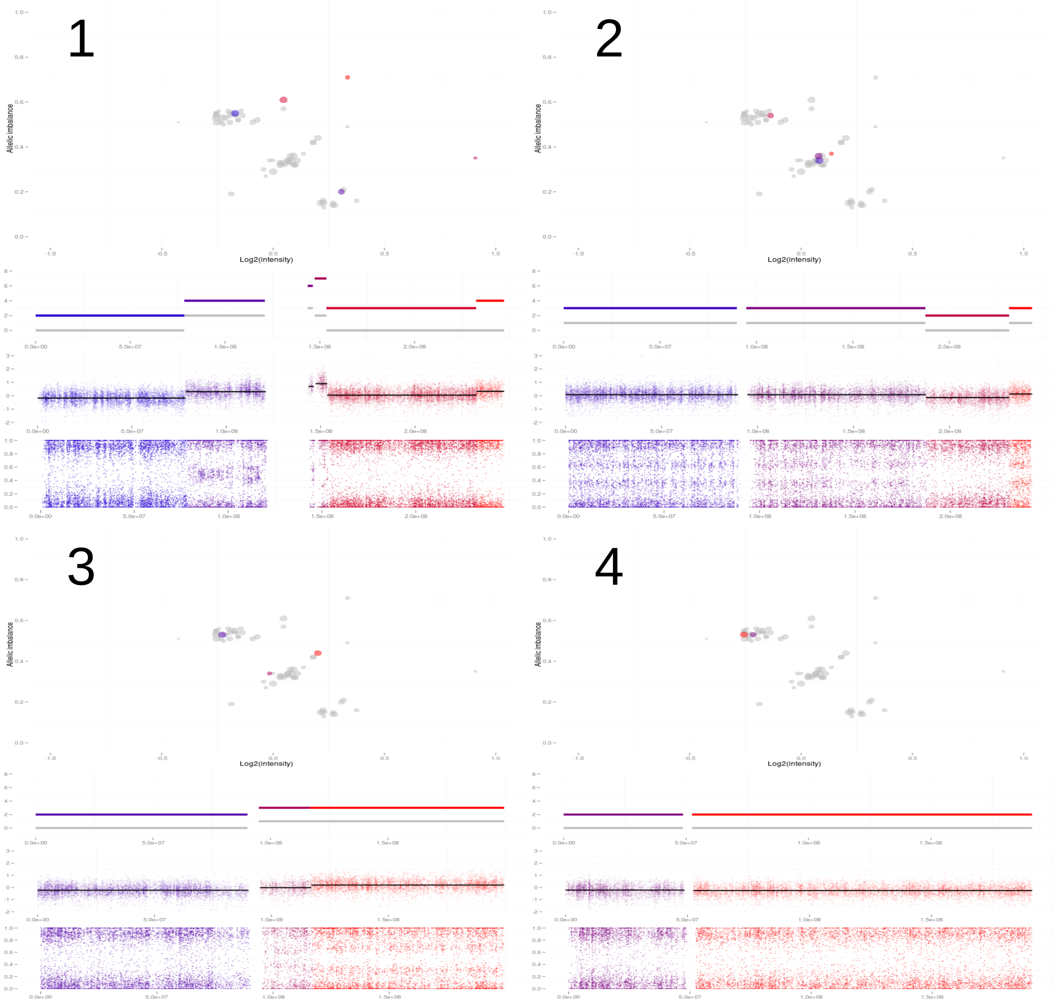

Lung cancer cell line H1437. TAPS scatter plots of chromosomes 1-4 display the segments of the current chromosome (color-coded blue to red) in relation to all other segments thoughout the sample (grey dots). The Log-ratio and Allelic Imbalance Ratio corresponding to allele-specific copy numbers estimated by TAPS is displayed in the summary plot below. Copy numbers correspond well to the SKY karyotype. Note that a part of chromosome 3 has fused with chromosome 14.

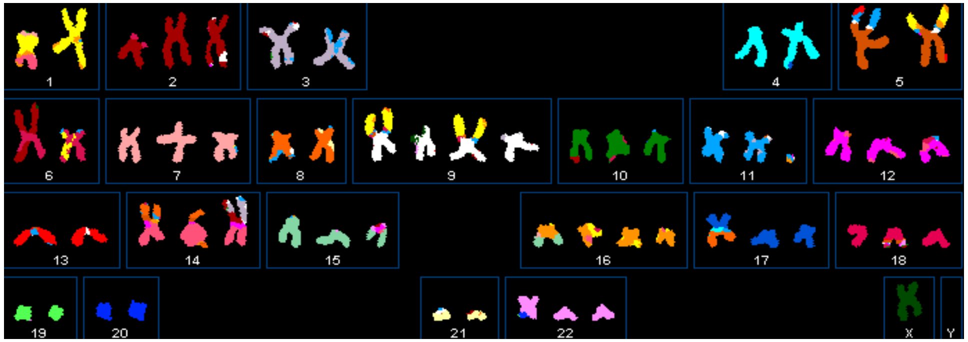

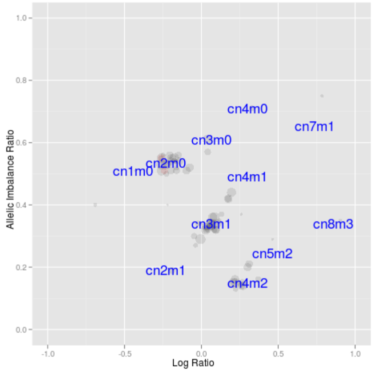

UPPSALA UNIVERSITET

H1770

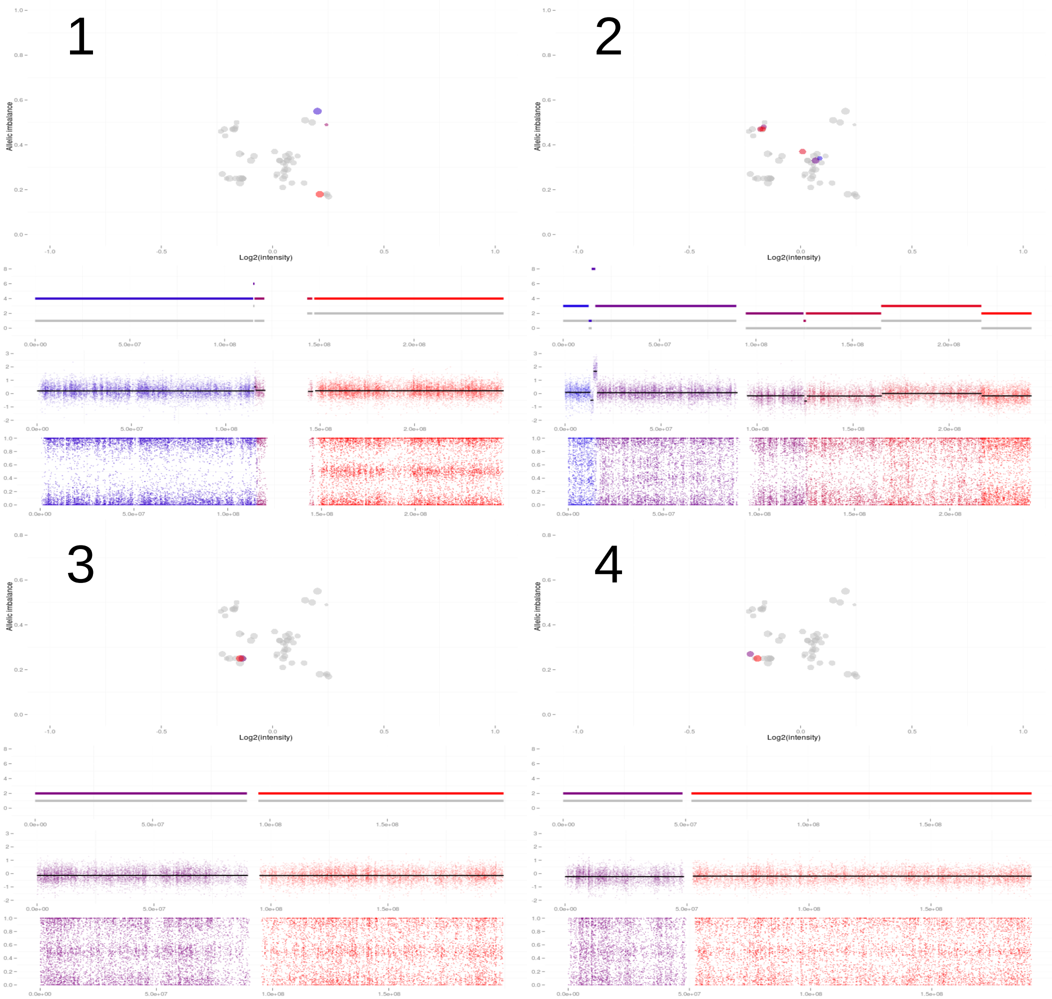

Lung cancer cell line H1770. TAPS scatter plots of chromosomes 1-4 display the segments of the current chromosome (color-coded blue to red) in relation to all other segments thoughout the sample (grey dots). The Log-ratio and Allelic Imbalance Ratio corresponding to allele-specific copy numbers estimated by TAPS is displayed in the summary plot below. Copy numbers correspond well to the SKY karyotype except for chromosome 4, which is obviously unaltered in the array experiment – note the highly balanced allele frequency.

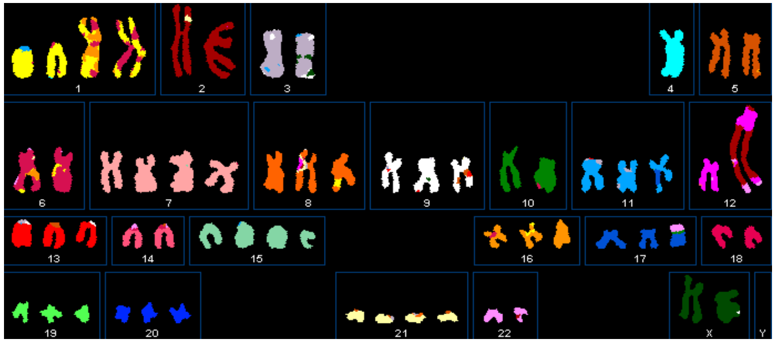

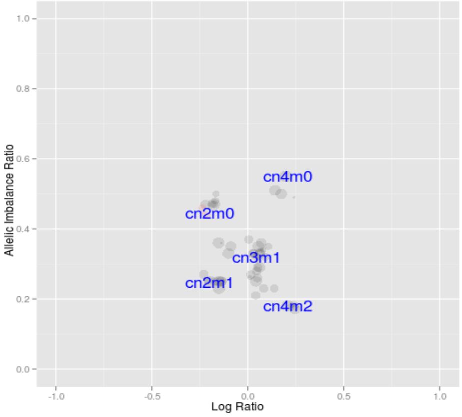

UPPSALA UNIVERSITET

H2087

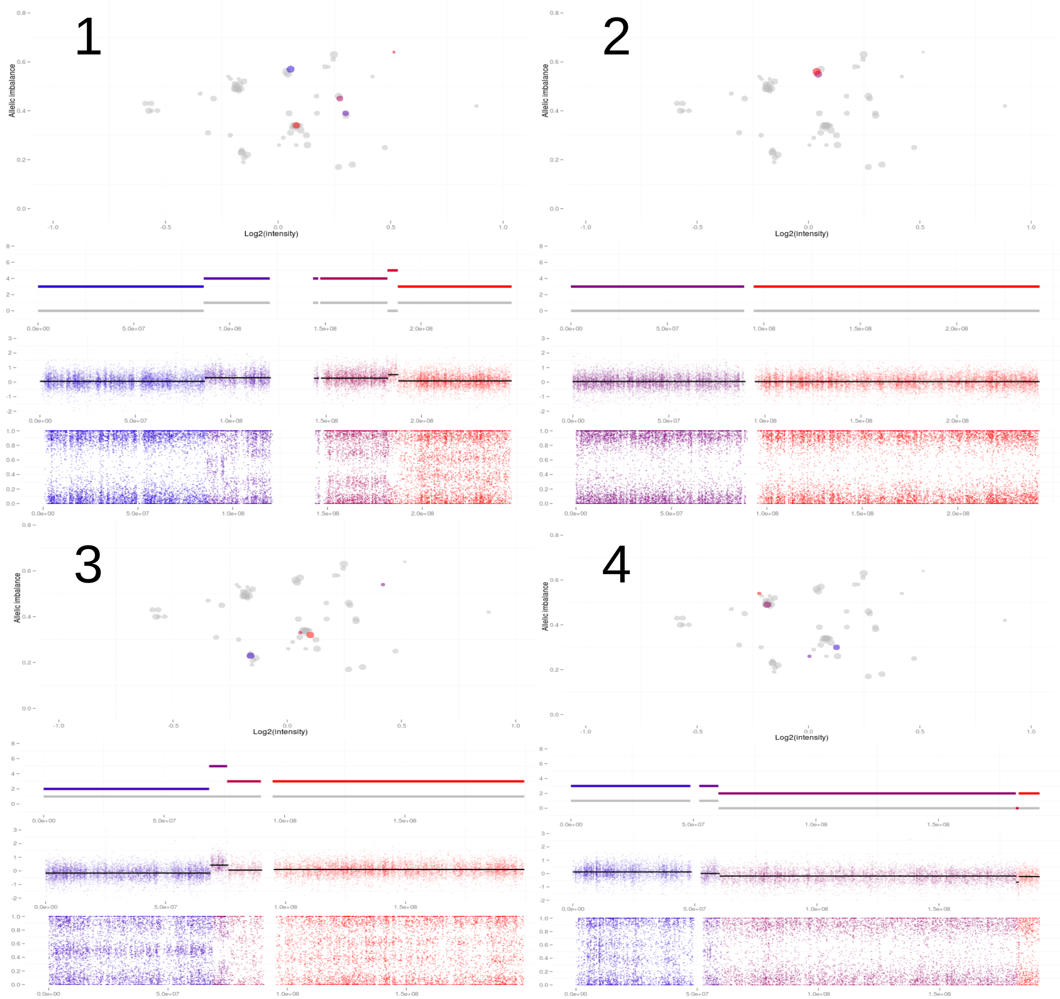

Lung cancer cell line H2087. TAPS scatter plots of chromosomes 1-4 display the segments of the current chromosome (color-coded blue to red) in relation to all other segments thoughout the sample (grey dots). The Log-ratio and Allelic Imbalance Ratio corresponding to allele-specific copy numbers estimated by TAPS is displayed in the summary plot below. Copy numbers correspond well to the SKY karyotype, although SKY imaging of chromosome 4 is absent.

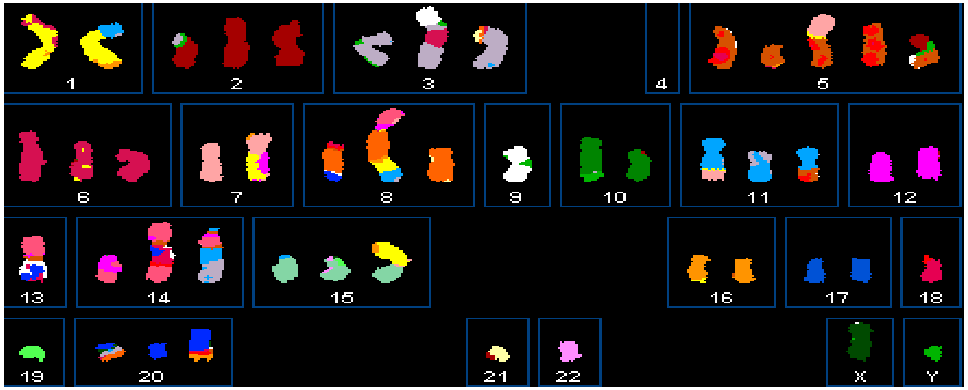

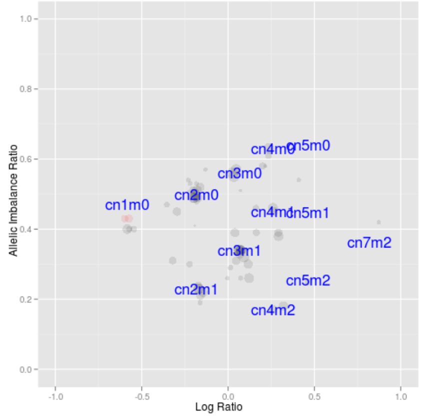

UPPSALA
UNIVERSITET

H2009

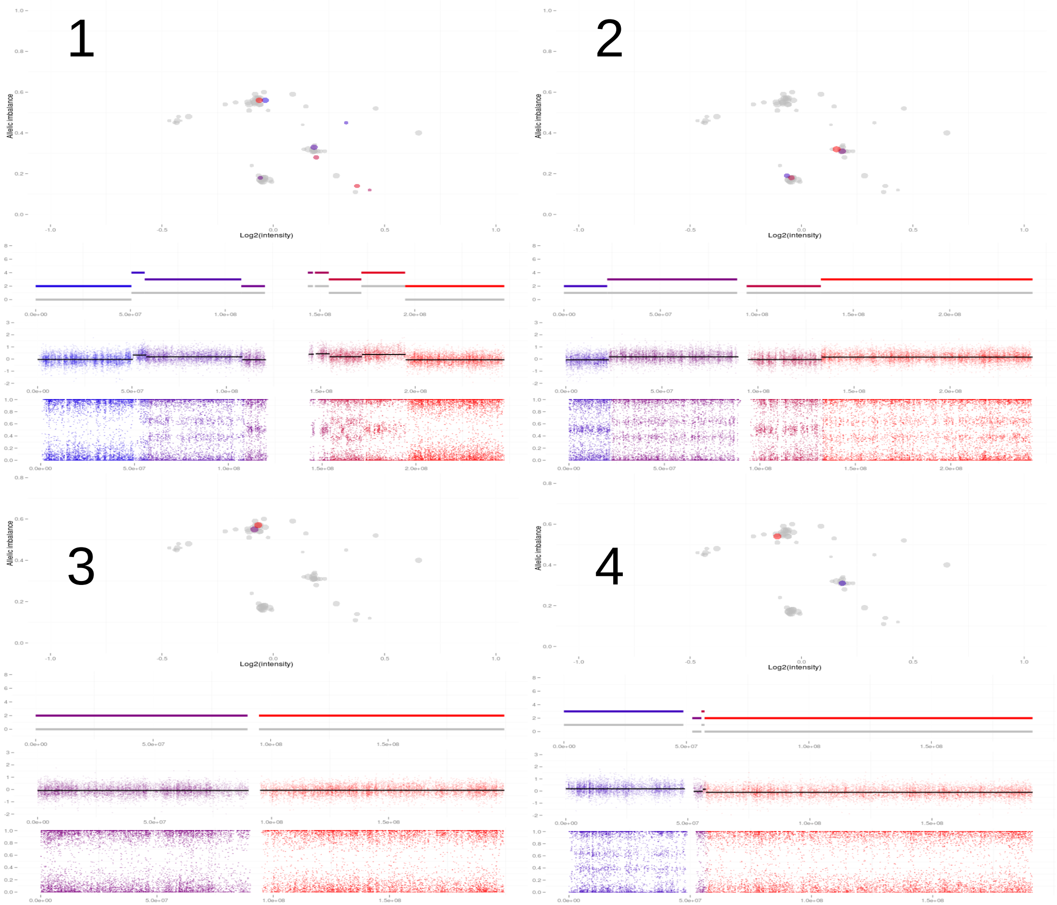

Lung cancer cell line H2009. TAPS scatter plots of chromosomes 1-4 display the segments of the current chromosome (color-coded blue to red) in relation to all other segments thoughout the sample (grey dots). The Log-ratio and Allelic Imbalance Ratio corresponding to allele-specific copy numbers estimated by TAPS is displayed in the summary plot below. Copy numbers correspond well to the SKY karyotype. Note that parts of chromosome 2 have fused with chromosomes 15 and 16.

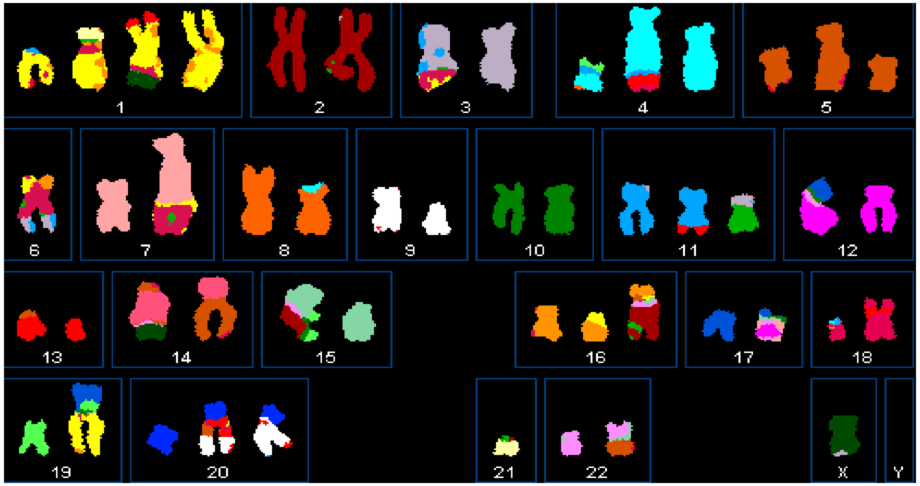

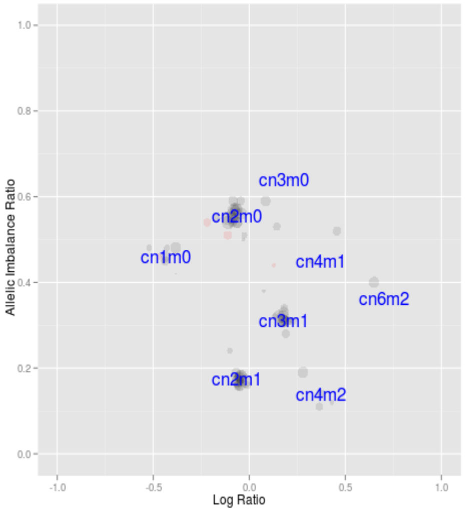

Supplement: Additional file 2 — Lung cancer cell line summaries. This file contains TAPS scatter plots illustrating the copy number analysis result of the seven lung cancer cell lines, and matching SKY karyotypes for comparison. [file gb-2011-12-10-r108-S2.PDF]
